# Linking MPV and NLR to TI-RADS: improved predictive accuracy for thyroid malignancy

**DOI:** 10.1097/MD.0000000000042452

**Published:** 2025-05-09

**Authors:** Ahmet Aydin, Sabin Goktas Aydin, Alper Cagri Karci

**Affiliations:** a Department of Internal Medicine, Medical Faculty, Istanbul Medipol University, Istanbul, Turkey; b Department of Medical Oncology, Istanbul SBU Kanuni Sultan Suleyman Training and Research Hospital, Istanbul, Turkey; c Department of Endocrinology, Istanbul Medipol University, Medical Faculty, Istanbul, Turkey.

**Keywords:** MPV, NLR, papillary thyroid cancer, thyroid nodule, TI-RADS

## Abstract

The incidence of papillary thyroid cancer has fluctuated, partly due to advancements in neck ultrasonography and fine-needle aspiration (FNA). Identifying additional markers to differentiate benign from malignant thyroid nodules could optimize patient management and reduce unnecessary procedures. This retrospective study included 355 patients categorized into those without nodules (group 1) and those with nodules (group 2). FNA results classified nodules as benign (group A) or malignant (group B). The Pearson and Spearman correlations, Student *t* test, Mann–Whitney *U* test, and receiver operating characteristic curve analysis calculated and compared inflammatory markers across groups. The study cohort included 126 patients without nodules (group 1), and 229 patients with nodules (group 2) of whom 39 were diagnosed with papillary thyroid cancer. The median age was 56, with 54.4% females and 45.6% males. Receiver operating characteristic analysis revealed significant but poor diagnostic performance for mean platelet volume (MPV) and neutrophil-to-lymphocyte ratio (NLR), with optimal cutoff values of 10.1 and 1.60, respectively (*P* < .001; area under the curve: 0.30), and *P* < .001, area under the curve: 0.24, respectively). Patients with MPV ≥ 10.1 fL had a higher prevalence of thyroid cancer (17.1%) compared to those with MPV < 10.1 fL (5.4%). Patients with NLR ≥ 1.6 exhibited a higher prevalence of thyroid cancer (54.7%) compared to those with NLR < 1.6 (4.6%). Higher MPV and NLR values were also significantly associated with higher thyroid imaging reporting and data system classifications (*P* < .001 and *P* = .05, respectively). Elevated MPV and NLR are significantly associated with thyroid cancer and higher thyroid imaging reporting and data system classifications. These markers, combined with ultrasonography and FNA, may aid in differentiating benign from malignant thyroid nodules, potentially improving patient management and reducing unnecessary procedures.

## 1. Introduction

Papillary thyroid cancer (PTC) is a differentiated epithelial-derived cancer. The incidence rose from 4.8 to 14.9 per 100,000, stabilized, and then appeared to decline to approximately 13.5 per 100,000 by 2018 based on the Surveillance, Epidemiology, and End Results database from 1975 to 2018.^[1,2]^ However, the main reason for this increase, apparent plateau, and subsequent decrease is unknown. The rise in reported cases of PTC is thought to be partially due to the increased use of neck ultrasonography (USG) and fine-needle aspiration (FNA) to detect smaller thyroid nodules at an earlier stage.^[3]^ PTC is more prevalent in females, with a female-to-male ratio of approximately 2.5:1, and the majority of cases occur in women during their 4^th^ and 5^th^ decades of life.^[4]^

Consistent with the rise in PTC, the incidence of thyroid nodules is also on the rise, and it is reported that it will affect 50% of the population by the age of 60.^[5]^ The underlying reason is the widespread use of high-resolution ultrasound, which has become the preferred method for detecting thyroid nodules combined with FNA.^[6]^ Thyroid imaging reporting and data system (TI-RADS) categorize thyroid nodules based on their features to assess their likelihood of being benign or malignant.^[7–9]^

While the majority of these nodules are noncancerous, they necessitate long-term monitoring, which can lead to psychological and economic strain on individuals.^[10]^ More judicious use of FNA in thyroid nodules and thyroid USG could decrease incident rates of unnecessary procedures. It is necessary to identify additional markers for predicting malign nodules. As a result, many studies recently have investigated the role of inflammation markers, such as neutrophil-to-lymphocyte ratio (NLR), platelet-to-lymphocyte ratio (PLR), and lymphocyte-to-monocyte ratio on thyroid nodules and malignant transformation.^[11–16]^ Additional markers, such as mean platelet volume (MPV), associated with functional changes in platelets, have also been studied in various types of cancer.^[17–19]^ However, none of the studies analyzed the association between inflammatory markers and TI-RADS combined with FNA.

Simple markers combined with TI-RADS should be determined, as this would become a common practice for diagnosing benign lesions and reducing invasive procedures. Concerning this knowledge, this study was conducted to explore further and validate potential laboratory parameters for helping differentiate benign and malignant thyroid nodules.

## 2. Materials and methods

### 2.1. Patient selection

The ultrasonographic data of 950 patients aged 18 to 65 who visited our hospital’s general internal medicine outpatient clinics with various symptoms between February 2021 and September 2024 and underwent neck USG were retrospectively screened. A total of 355 individuals were enrolled in the study.

Exclusion criteria included non-PTC histology, acute infection, thalassemia, iron deficiency with or without anemia, infectious diseases, connective tissue diseases, autoimmune diseases, hematological/oncological malignancies, diabetes mellitus, coronary artery disease, subclinical or clinical hypo/hyperthyroidism, subacute thyroiditis, renal or hepatic dysfunction, alcohol abuse, and pregnancy.

Patients were initially categorized into 2 groups: those without nodules (group 1) and those with nodules (group 2). According to American College of Radiology recommendations, patients who underwent FNA were classified as having benign (group A) or malignant nodules (group B).

The laboratory markers were collected from the antecubital vein and examined using the Mindray BC-6800 and ABBOT Architect 200-SR immunoassay analyzer. The NLR and PLR were computed using the following formulas: NLR = neutrophil count ÷ lymphocyte count; PLR = platelet count ÷ lymphocyte count. These ratios were then compared between the groups.

The Local Ethics Committee of Istanbul Medipol University granted ethical approval in May 2024 (decision number: E-10840098-202.3.02-3014). Written informed consent was obtained from all participants.

### 2.2. Ultrasound evaluation

The thyroid nodules were assessed using the American College of Radiology TI-RADS. The TI-RADS categories are as follows: TR1 corresponds to benign, TR2 to not suspicious, TR3 to probably benign, TR4 to suspicious for malignancy, and TR5 to highly suggestive of malignancy. Patients underwent FNA according to the recommendations based on TI-RADS, which were: TR1 requires no FNA, TR2 requires no FNA, TR3 suggests follow-up for nodules ≥ 1.5 cm, and FNA for nodules ≥ 2.5 cm, TR4 suggests follow-up for nodules ≥ 1.0 cm and FNA for nodules ≥ 1.5 cm, and TR5 suggests follow-up for nodules ≥ 0.5 cm and FNA for nodules ≥ 1.0 cm.^[8]^ The one with the highest TI-RADS score was selected for patients with multiple nodules, and the corresponding pathology was noted.

### 2.3. Statistical analysis

All statistical analyses were conducted using SPSS version 22.0 (SPSS Inc, Chicago). For data evaluation, alongside descriptive statistical techniques (including mean, standard deviation [SD], median, frequency, ratio, minimum, and maximum), the Shapiro–Wilk test was employed to assess data distribution. Pearson correlation was applied to data with a normal distribution, while Spearman correlation was used for data that did not follow a normal distribution. To compare quantitative data between 2 groups with normal distribution, the Student *t* test was utilized; for groups without normal distribution, the Mann–Whitney *U* test was used. In addition, a receiver operating characteristic (ROC) analysis was carried out to determine the optimal cutoff value for NLR and MPV. Statistical significance was defined as *P* < .05.

## 3. Results

### 3.1. Patients’ characteristics

The total number of patients was 355, with a median age of 56. A total of 54.4% of the patients were female, and 45.6% were male.

The patients were categorized into those without nodules (n = 126) and those with nodules (n = 226). FNA results classified nodules as benign (n = 316) or malignant (n = 39). The study cohort consisted of 3 groups: patients with no nodules (N = 126), patients with nodules detected on USG (N = 229), and patients diagnosed with PTC (N = 39).

The median age of patients was 53 (range: 36–75) in the no nodules group, 57 (range: 25–75) in the nodules on the USG group, and 54 (range: 46–69) in the PTC group. The gender distribution showed 84 males and 42 females in the no nodules group, 109 males and 120 females in the nodules on the ultrasound group, and 12 males and 27 females in the PTC group.

Among patients with nodules on USG, the distribution of TI-RADS classifications was as follows: TI-RADS 1 (n = 56), TI-RADS 2 (n = 29), TI-RADS 3 (n = 64), TI-RADS 4 (n = 54), and TI-RADS 5 (n = 26). In the PTC group, TI-RADS classifications were TI-RADS 3 (n = 3), TI-RADS 4 (n = 16), and TI-RADS 5 (n = 20), with no cases in TI-RADS 1 or 2.

The mean ± SD of MPV was 9.8 ± 0.92 in the no nodules group, 10.2 ± 0.84 in the nodules on ultrasound group, and 10.6 ± 0.87 in the PTC group. The mean ± SD of NLR was 1.67 ± 0.67 in the no nodules group, 1.78 ± 0.76 in the nodules on USG group, and 2.44 ± 1.04 in the PTC group (Supplementary Table S1, Supplemental Digital Content, https://links.lww.com/MD/O883). The mean ± SD of thyroid-stimulating hormone levels (U/L) was 1.2 ± 0.16 in the no nodules group, 1.5 ± 0.80 in the nodules on USG group, and 1.7 ± 0.82 in the PTC group.

Table 1 summarizes the patients’ characteristics.

**Table 1:** Patients’ characteristics.

Characteristic	Patients with		
	No nodules (N = 126)	Nodules on USG (N = 229)	Papillary thyroid cancer (N = 39)
TI-RADS			
1		56	–
2	–	29	–
3		64	3
4		54	16
5		26	20
Age years			
Median (range)	53 (36–75)	57 (25–75)	54 (46–69)
Gender (n)			
Male	84	109	12
Female	42	120	27
MPV (mean ± SD)	9.8 ± 0.92	10.2 ± 0.84	10.6 ± 0.87
NLR (mean ± SD)	1.67 ± 0.67	1.78 ± 0.76	2.44 ± 1.04
TSH (U/L) (mean ± SD)	1.2 ± 0.16	1.5 ± 0.80	1.7 ± 0.82

MPV = mean platelet volume, NLR = neutrophil-to-lymphocyte ratio, TI-RADS = thyroid imaging reporting and data system, TSH = thyroid-stimulating hormone, USG = ultrasonography.

### 3.2. Comparison between groups

The analysis of gender distribution revealed a potential association between female sex and thyroid cancer (*P* = .002). Within the cancer group, females comprised a significantly larger proportion (69.2%) than males (30.8%). Although females also constituted a slightly higher percentage of the overall study population (45.6% vs 54.4% males), this difference was not statistically significant.

This cross-tabulation analysis revealed a statistically significant association (*P* < .001) between gender and TI-RADS classification. While the overall distribution of genders across all TI-RADS categories remained relatively similar (males: 54.4% and females: 45.6%), a trend emerged when considering specific classifications. Females exhibited a higher prevalence in categories suggesting potentially more aggressive nodules (TI-RADS 3, 4, and 5). For instance, 62.5% of individuals with a TI-RADS classification of 3 were female, compared with 37.5% male. This pattern contrasted with lower TI-RADS classifications (1 and 2), where males represented a slightly larger proportion (around 59%).

No significant relationship was found between platelet count, whole blood cell count, hemoglobin level, thyroid-stimulating hormone, T levels, and a thyroid nodule or PTC. Age also did not significantly differ between groups (*P* > .05).

**Figure 1.**
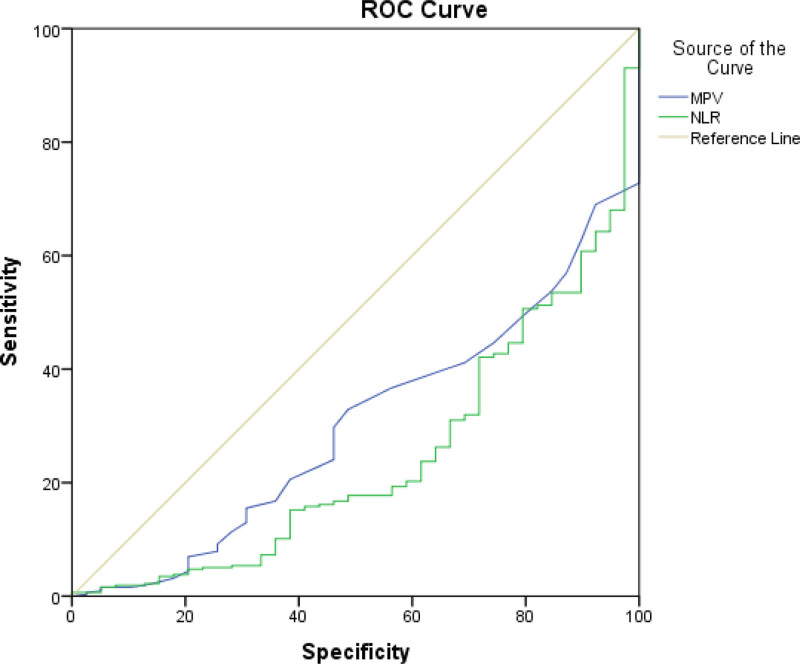
ROC curve of MPV and NLR predicting malign nodules. MPV = mean platelet volume, NLR = neutrophil-to-lymphocyte ratio, ROC = receiver operating characteristic.

There was a significant difference in MPV and NLR values between groups A and B (*P* = .05). The difference was also significant in groups 1 and 2, which demonstrated that MPV and NLR could predict malignancy and the presence of a thyroid nodule. The ROC curve analysis for the prediction of PTC is illustrated in Figure 1. The area under the curve (AUC) for MPV was 0.30 (95% confidence interval [CI]: 0.22–0.37, *P* < .001), indicating poor diagnostic performance. The optimal cutoff value for MPV was 10.1, yielding a sensitivity and specificity of 41% and 41%, respectively. For NLR, the AUC was 0.24 (95% CI: 0.16–0.32, *P* < .001), and the optimal cutoff value for NLR was identified as 1.60, with a sensitivity of 47% and a specificity of 31%.

**Figure 2.**
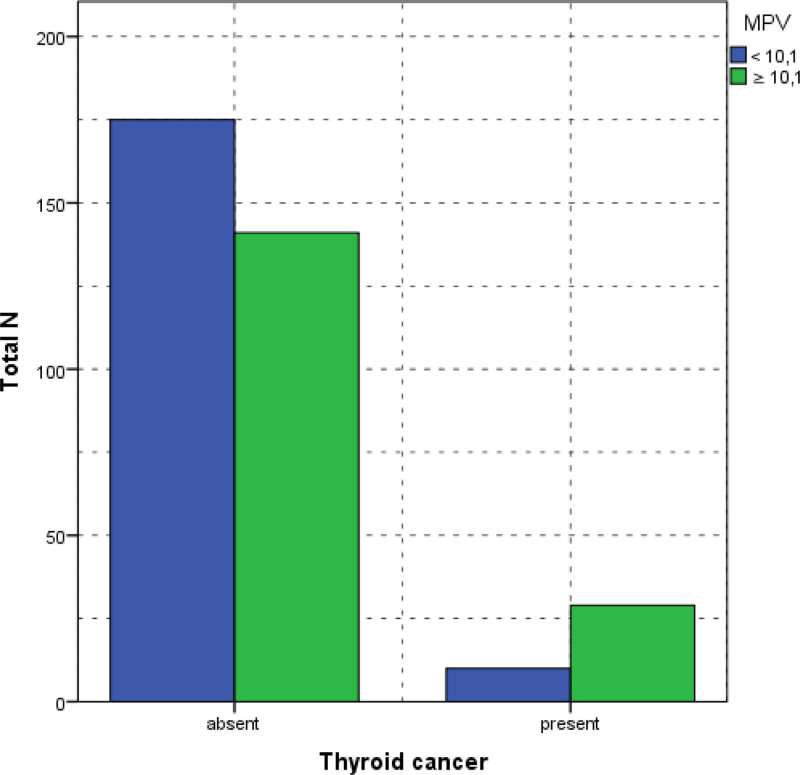
The comparison of NLR in patients with benign and malignant nodules. MPV = mean platelet volume, NLR = neutrophil-to-lymphocyte ratio.

**Figure 3.**
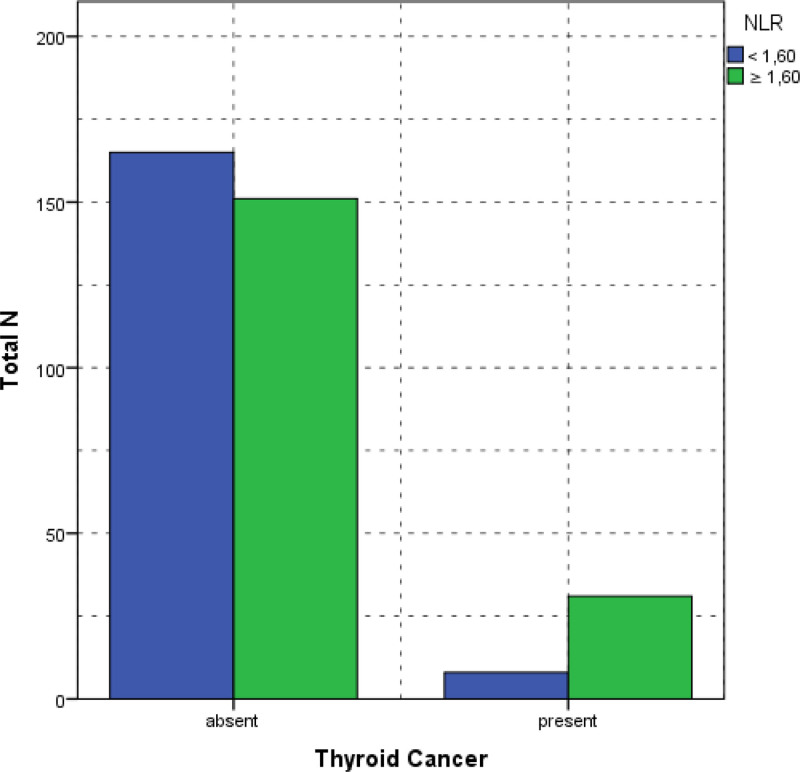
The comparison of MPV in patients with benign and malignant nodules. MPV = mean platelet volume, NLR = neutrophil-to-lymphocyte ratio.

This study revealed a statistically significant association between MPV and NLR with thyroid cancer (*P* = .001 and *P* < .001, respectively). Among individuals with MPV below 10.1 fL, only 10 (5.4%) presented with thyroid cancer, compared with 29 (17.1%) in the higher MPV group (≥10.1 fL) (Fig. 2). Similarly, patients with an NLR of <1.6 exhibited a significantly lower prevalence of thyroid cancer (4.6%) compared to those with an NLR of 1.6 or greater (54.7%) (Fig. 3).

Table 2 summarizes the distribution of patients with respect to MPV and NLR.

**Table 2:** Comparison of MPV and NLR between the patients with malign and benign nodules.

	Thyroid papillary cancer, n (%)		*P*
	Absent	Present	
MPV			.001
<10.1	175 (55.4%)	141 (44.6%)	
≥10.1	10 (25.6%)	29 (74.4%)	
NLR			<.001
<1.60	165 (52.2%)	151 (47.8%)	
≥1.60	8 (20.5%)	182 (51.3%)	

MPV = mean platelet volume, NLR = neutrophil-to-lymphocyte ratio.

TI-RADS classification according to MPV and NLR was evaluated. A statistically significant association emerged between MPV and TI-RADS scores (*P* < .001). Individuals with higher MPV (≥10.1 fL) exhibited a propensity for higher TI-RADS classifications compared to those with lower MPV (<10.1 fL). NLR also demonstrated a trend toward association with TI-RADS scores (*P* = .05). Patients with elevated NLR (≥1.60) presented a slightly greater prevalence of higher TI-RADS scores (Table 3).

**Table 3:** Comparison of MPV and NLR between the patients with no nodule and detected nodules on the USG.

	Control group	TI-RADS					*P*
		1	2	3	4	5	
MPV							.001
<10.1	85 (67.5)	25 (44.6)	12 (41.4)	27 (42.2)	30 (55.6)	6 (23.1)	
≥10.1	41 (32.5)	31 (55.4)	17 (58.6)	37 (57.8)	24 (44.4)	20 (76.9)	
NLR							.05
<1.60	67 (53.2)	32 (57.1)	13 (44.8)	34 (53.1)	19 (35.2)	8 (30.8)	
≥1.60	59 (46.8)	24 (42.9)	16 (55.2)	30 (46.9)	35 (64.89	18 (69.2)	

MPV = mean platelet volume, NLR = neutrophil-to-lymphocyte ratio, TI-RADS = thyroid imaging reporting and data system, USG = ultrasonography.

## 4. Discussion

NLR and PLR are well known for predicting inflammation. Another marker, MPV, an index of routine hemogram tests, shows the platelet size.^[16]^ Inflammation causes the activation of platelets, which results in increased MPV. The incidence of thyroid nodules is increasing, and reducing unnecessary interventions is crucial. On the other hand, inflammation marker tests are cost-effective and easy to use. Therefore, this study aimed to examine the role of inflammation in PTC, the most-seen endocrine cancer type, and the relationship between TI-RADS and inflammatory markers to predict malignancy in thyroid nodules.

Numerous studies on various cancer types have proved the role of inflammation in cancer development and prognosis. A recent systematic review by Detopoulou et al^[17]^ confirmed that elevated MPV was associated with adverse outcomes in several cancers, including breast, colorectal, gastric, lung, and pancreatic malignancies highlighting its potential role as a prognostic marker. Similarly, in a real-life cohort of advanced biliary tract cancer patients, Aydin et al^[18]^ demonstrated that elevated systemic inflammatory markers, particularly NLR and PLR, were significantly associated with overall survival, emphasizing their prognostic relevance.

Studies in thyroid cancer are controversial. Seretis et al^[20]^ first reported significantly elevated preoperative NLR in patients with papillary thyroid microcarcinomas. This was supported by Kim et al^[21]^ and Kocer et al,^[22]^ although Liu et al^[23]^ observed this elevation only in patients over 45. A subsequent meta-analysis did not confirm this relation with age.^[6]^ Haider et al^[24]^ studied the prediction of NLR in thyroid malignancy, which did not show a significant association. A significant prognostic role of NLR in PTC was proved by Treitsman et al^[25]^ However, Russo et al^[26]^ did not support these findings.

An optimized cutoff value of NLR is also essential. A study reported a logistic regression model revealed that an NLR > 1.49,529 serves as a prognostic indicator and an independent risk factor in pediatric thyroid cancer.^[27]^ In another study, a cutoff value 3 was significant for prognosis.^[25]^ Recently, Ge et al^[28]^ reported that NLR with a cutoff value of 1.845 (AUC: 0.612 ± 0.097) had a sensitivity of 60.0% and specificity of 66.2% (*P* = .027).

Our study suggested that NLR was a significant predictor of malign nodules, and as the TI-RADS score increased, a significant increase in the NLR level was observed. The ROC analysis demonstrated that a cutoff value of 1.60 for NLR had a sensitivity of 47% and a specificity of 31% (AUC: 0.24, 95% CI: 0.16–0.32, *P* < .001). Patients with NLR ≥ 1.6 exhibited a higher prevalence of thyroid cancer (54.7%) compared to those with NLR < 1.6 (4.6%) (*P* < 0,001). To summarize, NLR had a significant role in predicting the malignancy of thyroid nodules, but the cutoff had limited utility as a predictive marker for PTC in this cohort. Increasing the number of patients would be beneficial for optimizing the cutoff value.

Zhang et al^[15]^ were the first to assess the combination of TI-RADS grading with NLR in identifying the nature of thyroid nodules. They found that the proportion of malignant nodules with NLR ≥ 1.87 was significantly higher than those with NLR < 1.87 (*P* < .001) and that the proportion with K-TI-RADS grades ≥ 4a was significantly higher than those with K-TI-RADS grades < 4a (*P* < .001). This study differs from our investigation of using K-TI-RADS for differentiating malignant nodules. We examined the relationship between NLR and TI-RADS scores and whether NLR could distinguish between malignant and benign nodules.

In our analysis, patients with higher NLR values (≥1.60) were more frequently found in the higher TI-RADS categories. Specifically, 64.8% of those in TI-RADS 4 and 69.2% in TI-RADS 5 had elevated NLR, compared with a more even distribution in the lower categories. This pattern suggests that an increased NLR may be associated with a greater risk of malignancy, as reflected by higher TI-RADS scores. These findings highlight the potential value of incorporating NLR into existing risk stratification to help reduce unnecessary biopsies (*P* = .05).

Similarly to our study, Cheong et al^[29]^ investigated the diagnostic, predictive value of NLR in thyroid cancers and reported that preoperative NLR was significantly associated with adverse prognostic factors, such as larger tumor size and lateral lymph node metastasis. However, while our findings demonstrated that NLR and MPV were significantly elevated in malignant nodules across all TI-RADS categories and regardless of nodule size, Cheong et al ^[29]^ noted that NLR only served as a significant predictor in cases with nodules larger than 2 cm, losing predictive power in nodules under 2 cm. This difference might be partly attributed to study design variations; Cheong et al^[29]^ included a higher proportion of malignant cases (81.4% vs our 11%) and used a surgical cohort, which may have introduced selection bias toward more advanced disease.

MPV was linked with a poorer prognosis in various cancer types as well.^[17]^ However, the literature on MPV in thyroid malignancy is conflicting. Bayhan et al^[30]^ reported that MPV was significantly higher in patients with malignant thyroid diseases than benign ones. Sit et al^[12]^ supported these findings in 199 healthy individuals by showing that MPV significantly differed in the malignant and benign nodule groups (*P* < .001, 9.1 ± 1 vs 7.8 ± 0.8 fL respectively). Li et al^[31]^ studied the prognostic impact of MPV in medullary thyroid cancer. They showed that MPV ≤ 8.2 fL (odds ratio = 13.999) was an independent risk factor for calcitonin progression and significantly predicted lymph node metastasis. However, Bostan et al.^[32]^ and Kayataş et al^[11]^ did not demonstrate a significant relationship between MPV and malign thyroid nodules. Martin et al^[33]^ conducted a large retrospective study comparing preoperative inflammatory markers and platelet indices between 234 patients with PTC and 108 patients with benign thyroid pathology. They reported significantly higher platelet counts and platelet crit levels in malignant cases compared to benign ones. However, they did not observe any significant differences in MPV or NLR between groups.

In this study, MPV values significantly increased as the TRADS score increased (*P* < .001). In addition, MPV was considerably higher in the PTC group. In addition, a cutoff of 10.1 fL for MPV was achieved by ROC analysis, yielding sensitivity and specificity of 41% and 41%, respectively.

To our knowledge, this is the first study to assess the association between inflammatory markers and TI-RADS classification, combined with appropriate FNA, while also analyzing their relationship with malignancy. However, our study also presents certain limitations. The retrospective design and the relatively small sample size may affect the generalizability of our findings. In addition, the diagnostic performance of MPV and NLR, as indicated by the ROC analysis, showed poor sensitivity and specificity, limiting their standalone utility as predictive markers.

## 5. Conclusion

We demonstrated that both elevated MPV and NLR were significantly associated with malignant thyroid nodules and higher TI-RADS scores. We identified a cutoff value of 10.1 fL for MPV and 1.60 for NLR, with both markers showing a clear correlation with malignancy. Despite modest diagnostic accuracy regarding AUC values (0.30 for MPV and 0.24 for NLR), combining these markers with TI-RADS scoring appeared to enhance risk stratification in our cohort.

Although future research with larger prospective cohorts is necessary to refine these markers’ cutoff values and validate their clinical applicability, this combined approach, inflammatory markers, when used alongside USG, may optimize patient management by potentially reducing unnecessary procedures, thereby alleviating the psychological and economic burden on patients.

In conclusion, our findings contribute to the growing body of literature exploring cost-effective and easily accessible markers for thyroid nodule assessment, encouraging further investigation into their integration with established diagnostic protocols.

## Author contributions

**Conceptualization:** Ahmet Aydin, Sabin Goktas Aydin.

**Data curation:** Ahmet Aydin, Alper Cagri Karci.

**Resources:** Ahmet Aydin.

**Supervision:** Ahmet Aydin, Alper Cagri Karci.

**Writing – original draft:** Ahmet Aydin, Alper Cagri Karci.

**Writing – review & editing:** Ahmet Aydin, Sabin Goktas Aydin, Alper Cagri Karci.

**Formal analysis:** Sabin Goktas Aydin.

**Methodology:** Sabin Goktas Aydin.

**Visualization:** Alper Cagri Karci.

## Supplementary Material



## References

[1] NIH. Cancer stat facts: thyroid cancer. http://seer.cancer.gov/statfacts/html/thyro.html. Accessed April 14, 2022.

[2] Powers AE Marcadis AR Lee M Morris LGT Marti JL. Changes in trends in thyroid cancer incidence in the United States, 1992 to 2016. JAMA. 2019;322:2440–1. 31860035 10.1001/jama.2019.18528 PMC6990659

[3] Vaccarella S Franceschi S Bray F Wild CP Plummer M Dal Maso L. Worldwide thyroid-cancer epidemic? The increasing impact of overdiagnosis. N Engl J Med. 2016;375:614–7. 27532827 10.1056/NEJMp1604412

[4] Davies L Welch HG. Current thyroid cancer trends in the United States. JAMA Otolaryngol Head Neck Surg. 2014;140:317–22. 24557566 10.1001/jamaoto.2014.1

[5] American Thyroid Association. Clinical thyroidology for the public. Thyroid nodules. 2018. https://www.thyroid.org/patient-thyroid-information/ct-for-patients/july-2018/vol-11-issue-7-p-11-12/. Accessed December 2, 2024.

[6] Trimboli P Castellana M Virili C. Performance of contrast-enhanced ultrasound (CEUS) in assessing thyroid nodules: a systematic review and meta-analysis using histological standard of reference. Radiol Med. 2020;125:406–15. 31970579 10.1007/s11547-019-01129-2

[7] Burman KD Wartofsky L. Clinical practice. Thyroid nodules. N Engl J Med. 2015;373:2347–56. 26650154 10.1056/NEJMcp1415786

[8] Shayganfar A Hashemi P Esfahani MM Ghanei AM Moghadam NA Ebrahimian S. Prediction of thyroid nodule malignancy using thyroid imaging reporting and data system (TI-RADS) and nodule size. Clin Imaging. 2020;60:222–7. 31927498 10.1016/j.clinimag.2019.10.004

[9] Alexander EK Cibas ES. Diagnosis of thyroid nodules. Lancet Diabetes Endocrinol. 2022;10:533–9. 35752200 10.1016/S2213-8587(22)00101-2

[10] Pandeya N McLeod DS Balasubramaniam K. Increasing thyroid cancer incidence in Queensland, Australia 1982-2008 – true increase or overdiagnosis? Clin Endocrinol (Oxf). 2016;84:257–64. 25597380 10.1111/cen.12724

[11] Kayataş K Taşçi ES Yildirim M. The relationship between red cell distribution width, mean platelet volume, neutrophil to lymphocyte ratio and benign thyroid nodules. Age (years). 2020;49:11–66.

[12] Sit M Aktas G Ozer B. Mean platelet volume: an overlooked herald of malignant thyroid nodules. Acta Clin Croat. 2019;58:417–20. 31969752 10.20471/acc.2019.58.03.03 PMC6971808

[13] Li C Li J Li S. Prognostic significance of inflammatory markers LMR, PLR, MPV, FIB in intermediate-and high-risk papillary thyroid carcinoma. Front Endocrinol (Lausanne). 2022;13:984157. 36060974 10.3389/fendo.2022.984157 PMC9434795

[14] Deng Y Zhang J Zou G. Peripheral blood inflammatory markers can predict benign and malignant thyroid nodules. Int J Endocrinol. 2022;2022:2319660. 35795844 10.1155/2022/2319660 PMC9251144

[15] Zhang J Gong Z Li S. The value of neutrophil-to-lymphocyte ratio combined with the thyroid imaging reporting and data system in diagnosing the nature of thyroid nodules. J Clin Lab Anal. 2022;36:e24429. 35403307 10.1002/jcla.24429 PMC9102493

[16] Liu JF Ba L Lv H. Association between neutrophil-to-lymphocyte ratio and differentiated thyroid cancer: a meta-analysis. Sci Rep. 2016;6:38551. 27941815 10.1038/srep38551 PMC5150572

[17] Detopoulou P Panoutsopoulos GI Mantoglou M. Relation of mean platelet volume (MPV) with cancer: a systematic review with a focus on disease outcome on twelve types of cancer. Curr Oncol. 2023;30:3391–420. 36975471 10.3390/curroncol30030258 PMC10047416

[18] Aydin SG Demirel BC Bilici A. Real-life analysis of treatment approaches and the role of inflammatory markers on survival in patients with advanced biliary tract cancer. Curr Med Res Opin. 2022;38:1751–8. 35916475 10.1080/03007995.2022.2108619

[19] Jung SH Hao J Shivakumar M. Development and validation of a novel strong prognostic index for colon cancer through a robust combination of laboratory features for systemic inflammation: a prognostic immune nutritional index. Br J Cancer. 2022;126:1539–47. 35249104 10.1038/s41416-022-01767-w PMC9130221

[20] Seretis C Gourgiotis S Gemenetzis G Seretis F Lagoudianakis E Dimitrakopoulos G. The significance of neutrophil/lymphocyte ratio as a possible marker of underlying papillary microcarcinomas in thyroidal goiters: a pilot study. Am J Surg. 2013;205:691–6. 23388425 10.1016/j.amjsurg.2012.08.006

[21] Kim JY Park T Jeong SH. Prognostic importance of baseline neutrophil to lymphocyte ratio in patients with advanced papillary thyroid carcinomas. Endocrine. 2014;46:526–31. 24272600 10.1007/s12020-013-0089-6

[22] Kocer D Karakukcu C Karaman H Gokay F Bayram F. May the neutrophil/lymphocyte ratio be a predictor in the differentiation of different thyroid disorders? Asian Pac J Cancer Prev. 2015;16:3875–9. 25987053 10.7314/apjcp.2015.16.9.3875

[23] Liu J Du J Fan J. The neutrophil-to-lymphocyte ratio correlates with age in patients with papillary thyroid carcinoma. ORL J Otorhinolaryngol Relat Spec. 2015;77:109–16. 25896501 10.1159/000375534

[24] Haider N Mahmood Z Khalid F Razzak SA. Neutrophils to lymphocytes ratio between benign and malignant thyroid nodule. Pak J Med Sci. 2021;37:1908–11. 34912416 10.12669/pjms.37.7.4503 PMC8613057

[25] Treistman N Cavalcante LBCP Gonzalez F. Neutrophil-to-lymphocyte ratio as an independent factor for worse prognosis in radioiodine refractory thyroid cancer patients. Endocrine. 2023;81:141–8. 36905576 10.1007/s12020-023-03340-8

[26] Russo E Guizzardi M Canali L. Preoperative systemic inflammatory markers as prognostic factors in differentiated thyroid cancer: a systematic review and meta-analysis. Rev Endocr Metab Disord. 2023;24:1205–16. Erratum in: Rev Endocr Metab Disord. 2023 Nov 18. 37828383 10.1007/s11154-023-09845-x

[27] Li S Liu Y Liu S Du G Wang Z Yin D. Predictive values of inflammation-related markers and thyroid function in pediatric thyroid cancer patients. Front Pediatr. 2021;9:802214. 35004550 10.3389/fped.2021.802214 PMC8740167

[28] Ge J Guo X Zhao W. Evaluation of pre-ablation NLR and LMR as predictors of distant metastases in patients with differentiated thyroid cancer. Acta Endocrinol (Buchar). 2023;19:215–20. 37908873 10.4183/aeb.2023.215 PMC10614579

[29] Cheong TY Hong SD Jung KW So YK. The diagnostic predictive value of neutrophil-to-lymphocyte ratio in thyroid cancer adjusted for tumor size. PLoS One. 2021;16:e0251446. 33974674 10.1371/journal.pone.0251446 PMC8112685

[30] Bayhan Z Zeren S Ozbay I. Mean platelet volume as a biomarker for thyroid carcinoma. Int Surg. 2016;101:50–3. 10.9738/INTSURG-D-15-00123.1 26160507

[31] Li C Zhang H Li S. Prognostic impact of inflammatory markers PLR, LMR, PDW, MPV in medullary thyroid carcinoma. Front Endocrinol (Lausanne). 2022;13:861869. 35350101 10.3389/fendo.2022.861869 PMC8957807

[32] Bostan H Sencar ME Calapkulu M. The predictive value of hematologic parameters in the risk of thyroid malignancy in cases with atypia/follicular lesion of undetermined significance. Eur Arch Otorhinolaryngol. 2022;279:4077–84. 35006341 10.1007/s00405-021-07248-9

[33] Martin S Mustata T Enache O. Platelet activation and inflammation in patients with papillary thyroid cancer. Diagnostics (Basel). 2021;11:1959. 34829306 10.3390/diagnostics11111959 PMC8624142

