# Supplementary material for: Linking MPV and NLR to TI-RADS: improved predictive accuracy for thyroid malignancy
Source: Medicine (Baltimore). 2025 May 9;104(19):e42452. doi: 10.1097/MD.0000000000042452 (PMC12074102; doi:10.1097/MD.0000000000042452)
Supplement: Supplementary file 1 [file medi-104-e42452-s001.docx]

| **Group** | **n** | **MPV (Mean ± SD)** | **NLR (Mean ± SD)** |
| --- | --- | --- | --- |
| No Nodules (Group 1) | 126 | 9.8 ± 0.92 fL | 1.67 ± 0.67 |
| Nodules on USG (Group 2) | 229 | 10.2 ± 0.84 fL | 1.78 ± 0.76 |
| PTC (Group B) | 39 | 10.6 ± 0.87 fL | 2.44 ± 1.04 |

Supplementary Table 1: The mean values of NLR and MPV per group
